# Instrumental learning in social interactions: Trait learning from faces and voices

**DOI:** 10.1177/1747021821999663

**Published:** 2021-03-21

**Authors:** Abigail R Bradshaw, Carolyn McGettigan

**Affiliations:** 1 Department of Speech, Hearing and Phonetic Sciences, University College London, London, UK; 2 Department of Experimental Psychology, University of Oxford, Oxford, UK

**Keywords:** Voices, faces, traits, reinforcement learning, social interaction

## Abstract

Recent research suggests that reinforcement learning may underlie trait formation in social interactions with faces. The current study investigated whether the same learning mechanisms could be engaged for trait learning from voices. On each trial of a training phase, participants (*N* = 192) chose from pairs of human or slot machine targets that varied in the (1) reward value and (2) generosity of their payouts. Targets were either auditory (voices or tones; Experiment 1) or visual (faces or icons; Experiment 2) and were presented sequentially before payout feedback. A test phase measured participant choice behaviour, and a post-test recorded their target preference ratings. For auditory targets, we found a significant effect of reward only on target choices, but saw higher preference ratings for more generous humans and slot machines. For visual targets, findings from previous studies were replicated: participants learned about both generosity and reward, but generosity was prioritised in the human condition. These findings provide one of the first demonstrations of reinforcement learning of reward with auditory stimuli in a social learning task, but suggest that the use of auditory targets does alter learning in this paradigm. Conversely, reinforcement learning of reward and trait information with visual stimuli remains intact even when sequential presentation introduces a delay in feedback.

Faces and voices are important social stimuli that play a key role in social cognition during interpersonal interactions (Hassin & Trope, 2000). For example, these stimuli can be mapped onto representations of abstract concepts such as traits and attitudes. The attribution of traits to social identities is essential in guiding appropriate behaviour in social interactions. It can be used to guide predictions of the future behaviour of social partners, as well as our own decisions about how and whether to interact with that partner in future (Eysenck, 1947; Heider, 1958; Jones & Davis, 1965). Crucially, the attribution of traits to a social identity is consistent across contexts; while the reward value of any one particular interaction with a social partner may vary, traits are assumed to be stable across contexts (Heider, 1944). For example, we may be likely to continue to pursue interactions with a social partner who is perceived as generous, even if the last time we met them they had forgotten their wallet.

There is evidence that people rapidly form judgements of personality traits from mere exposure to new voices and faces, without observation of any behaviour. Multiple studies have shown that people form trait impressions from briefly presented static images of unfamiliar faces (Todorov et al., 2009) and from brief utterances spoken by novel voices (McAleer et al., 2014). Furthermore, these rapid trait attributions can be consistent across viewers/listeners (McAleer et al., 2014; Todorov et al., 2008). Such findings have been used to argue that these “first impressions” may be based on consistent physical characteristics that have evolutionary significance, such as cues to health or reproductive success (Little et al., 2011; Pisanski & Feinberg, 2018; Puts et al., 2012; Zebrowitz & Montepare, 2008). Other work, however, has reported reliable individual differences in these rapid trait impressions, which were explained by variation in individual experience, rather than genetic influences (Germine et al., 2015; Sutherland et al., 2020).

In addition to this process of “reading from faces” (in which facial features affect trait impressions), the complementary process of “reading into faces” (in which trait impressions can change perception of facial features) has also been reported (Hassin & Trope, 2000). Facial appearance can also affect trait inferences through the process of stimulus generalisation; individuals have been shown to distrust strangers who implicitly resemble others they know to be untrustworthy, but trust strangers with facial features that resemble those they know to be trustworthy (Feldman-Hall et al., 2018). Rapid trait impressions can also affect responses towards those individuals; perceived facial and vocal personality from short exposures can affect voting behaviour, mate selection, and criminal conviction decisions (Chen et al., 2016; Klofstad et al., 2012; Mileva et al., 2020; Tigue et al., 2012; Zebrowitz & McDonald, 1991).

However, in addition to these rapid judgements of personality, it is adaptive for people to learn about the traits of social partners through observation of their behaviour. This raises the question of whether one can use interactions with face and voice stimuli to train individuals to attribute certain personality traits to a social identity. This has real-world significance for technologies that use voices to represent artificial agents, for example, mobile phone virtual assistants. In such cases, it would clearly be beneficial to use voices that are perceived as having positive personality traits, such as trustworthiness. Indeed, there is evidence that manipulating the physical characteristics of voices (e.g., expression of emotion) can affect participants’ perception of personality traits in artificial agents (Torre et al., 2020). However, we can further ask if it is possible to train individuals to attribute positive or negative traits to different voice or face identities based on experience of their behaviour in interactions.

Previously, the majority of work on manipulating trait formation has focused on the inference of traits through instruction or observational learning; for example, through reading of descriptions of a person’s behaviour designed to imply specific traits, for example, trustworthiness (Lavan et al., 2020; Rim et al., 2009). More recent work, however, has considered how trait impressions can be formed through feedback-based reinforcement learning. For example, a paradigm known as the Trust game (Berg et al., 1995) has been used to investigate how participants learn about the trustworthiness of partners. In a series of interactions, participants choose how much money to invest in a partner (providing a measure of trust); this amount is multiplied by a factor set by the experimenter, and the participant is then told how much of this larger amount their partner decided to share back with them (providing feedback on the partner’s trustworthiness). Participants are more like to place trust (i.e., invest) in partners who have previously reciprocated trust (i.e., who return more money than was initially invested) (King-Casas et al., 2005). Furthermore, a study by Chang et al. (2010) found that initial implicit trustworthiness judgements of the faces representing partners interacted with subsequent experienced trustworthiness when interacting with those partners in the Trust Game; partners who were initially judged as trustworthy and then behaved in a trustworthy manner were invested in the most. Thus, both implicit rapid trait impressions and experience of the behaviour of social partners can affect trust behaviour.

Other work has considered how such trait learning in reinforcement learning paradigms can be affected by the socialness of the learning context. This was investigated in the context of generosity trait inferences by Hackel et al. (2015). In this task, participants interacted with pairs of target identities, which shared different amounts of points with them over a series of trials. The participants’ instruction was to maximise their winnings in the game by selecting their preferred target on each trial. The behaviour of these targets was fixed to involve different levels of average reward (absolute number of points shared) and average generosity (relative number of points shared out of the total point pool available). These targets were posed either as other participants (represented by face stimuli) or as slot machines (represented by schematic pictures). When the interaction was framed as social, participants’ learning was biased towards trait information (i.e., they showed a preference for more generous human targets); conversely, when framed as non-social, learning was biased towards reward information (i.e., they showed a preference for more rewarding slot machine targets). The findings from this within-subjects design were later replicated in a between-subjects design by Hackel et al. (2020), this time using an identical set of visual fractal stimuli to represent either human or slot machine targets. This work demonstrates that the dynamics of instrumental learning can be changed by the socialness of the context, and provides evidence that reinforcement learning mechanisms may support the formation of trait perceptions in real-life social interactions.

To evaluate the extent to which reinforcement learning provides a good model of trait formation in social interactions, it is important to demonstrate that this type of learning also occurs for other types of social stimuli, such as voices. Indeed, associative learning with auditory stimuli in general, whether verbal or non-verbal, remains largely unexplored in the reinforcement learning literature. Therefore, an important outstanding question concerns whether participants are able to associate different reward and trait outcomes with different auditory identities through reinforcement learning. We can then further ask whether, as has been shown for visual stimuli, this learning is affected by the socialness of the framing context.

In Experiment 1 of the current study, we adjusted the paradigm used by Hackel et al. (2015, 2020) for use with auditory stimuli, to investigate these questions. The key adjustment to the original paradigm—a necessity for the use of auditory targets—was that pairs of stimuli on each trial were presented sequentially, rather than simultaneously. We predicted that participants would learn to associate differing levels of reward and generosity with different auditory identities, but that there would be a prioritisation of trait over reward information when those identities were presented as human (with voice stimuli), and vice versa when presented as non-human (with tone sequence “slot machine” stimuli). In Experiment 2, we used the same paradigm reported in Experiment 1 but now with visual stimuli (faces and schematic icons of slot machines), to investigate whether the pattern of learning reported in the studies by Hackel et al. could be demonstrated with sequential presentation of visual targets.

## Experiment 1: trait learning with auditory targets

### Method

This study was pre-registered on Open Science Framework prior to data collection (see https://osf.io/x93ng for pre-registration form). All deviations from this pre-registered protocol are outlined in the text below.

#### Participants

A total of 114 participants were recruited for this experiment through the online recruitment platform Prolific (www.prolific.ac). The Gorilla Experiment Builder (www.gorilla.sc) was used to create and host our experiment (Anwyl-Irvine et al., 2020). All participants underwent a headphone screening task, to ascertain that they were wearing headphones and listening in a quiet environment (Woods et al., 2017). Participants who failed to reach criterion performance on this task (score of at least 10/12) were not permitted to proceed to the main study. Data from 18 participants were excluded due to a failure to pass subsequent attention checks embedded in the tasks or to adhere to task instructions such as taking too long a break in between the tasks (see section “Data exclusion” for full description of exclusion criteria). After these exclusions, replacement participants were recruited to reach the target sample size of 96 participants (34 female, 61 male, 1 non-disclosed, mean age = 27.45, *SD* = 5.89). An equal number of participants took part in the two main conditions (48 in the human group, 48 in the non-human group).

Determination of sample size was guided by the sample size used in a previous study by Hackel et al. (2015) in which 30 participants completed both the human and the non-human conditions in a within-subjects design. Since effects could be weaker with voices (e.g., due to voice recognition being more error-prone than face recognition, see Stevenage et al., 2011) and the fact that online testing might involve noisier participant behaviour, we increased our target sample size from 30 to 48 participants per main condition; thus, we tested a total of 96 participants in our between-subjects design. We note here that a sample size of 96 deviates from our pre-registered sample size of 48—this figure was pre-registered in error, due to a simple oversight where we failed to account for the fact that our between-subjects design required two groups of independent participants, and so double the number of participants used in a within-subjects design to reach the same number of observations per condition.

This study received ethical approval from the local ethics officer at the Department of Speech, Hearing and Phonetic Sciences at University College London (approval no. SHaPS-2018-CM-029). All participants gave informed consent prior to taking part in the study.

#### Stimuli

Auditory stimuli consisted of four human voice clips (representing four human identities) and four tone sequence clips (representing four slot machine “identities”). The voice clips consisted of recordings of the word “hello” spoken in a neutral tone by four different male Southern Standard British English speakers. These were taken from a larger set of recordings collected for use in a different study (Payne et al., 2020). Selection of these voice clips from this larger pool was guided by participant ratings of different traits in previous pilot work carried out online. Twenty UK participants (11 female, age range of 19–41 years) provided ratings for 12 different voices using a 7-point Likert-type scale, in response to questions of the form “How attractive/likeable/trustworthy does the speaker sound?” We then selected four of these voices that were matched on these ratings of attractiveness, likeability, and trustworthiness (see Table 1 for mean ratings). The slot machine sounds consisted of short sequences of tones designed to be discriminable but similarly salient. All recordings (voices and tone sequences) were matched for token duration (around 400 ms) and sound intensity (via RMS-norming). Participants only ever encountered the voice stimuli (human group) or the tone-sequence stimuli (slot machine group). The same four tokens (one for each vocal identity/slot machine) were used throughout the whole experiment. These tokens are available online on OSF (https://osf.io/yx3jt/).

**Table 1.** Mean (*SD*) ratings on attractiveness, likeability, and trustworthiness for the four human voices.

Voice	Attractiveness	Likeability	Trustworthiness
Voice 1	3.90 (1.37)	5.21 (0.98)	5.15 (1.23)
Voice 2	3.85 (1.31)	5.00 (1.41)	5.15 (1.27)
Voice 3	3.95 (1.61)	5.10 (1.37)	5.35 (1.35)
Voice 4	4.30 (1.49)	5.15 (1.39)	5.15 (1.23)

These voice and tone sequence clips were accompanied by visual stimuli, which represented the location of each identity on the screen. Identical pictures (yellow loudspeaker icons) were used to represent each identity. These pictures pulsated when their corresponding auditory stimulus was played, to indicate the onscreen position (left/right) of each identity on that trial.

#### Procedure

Participants completed a reinforcement learning task closely based on that described in Hackel et al. (2015). In this task, participants learnt about the generosity and reward values associated with four different targets; either four human identities (represented by the voice stimuli) or four slot machines (represented by the tone sequence stimuli). In a between-subjects design, participants were randomly allocated to either the human voice target group or the non-human slot machine target group. Participants in the human group were told that they would have to learn about four previous Prolific participants who had made a series of choices about how to divide up a pool of points between themselves and the participant. Participants in the slot machine group were told they would have to learn about four computerised slot machines which were used to determine how many points to pay out to a participant from pools created by the experimenters. Other than these differences in instructions and in the auditory stimuli used, all other aspects of the study design and procedure were kept identical between the two conditions. Participants were told at the start of the game that they would be awarded a bonus payment based on the number of points they won; however, all participants were in fact paid the same fixed bonus amount at the end of the study that corresponded to the maximum amount they could have won.

Each of the four human/slot machine targets were assigned to one of four different generosity/reward conditions: high generosity, high reward; high generosity, low reward; low generosity, high reward; low generosity, low reward. Assignment of the targets to conditions was counterbalanced across participants. A total of 24 counterbalancing orders were possible; two participants from each group (human and non-human) were therefore randomly assigned to each order. The four conditions were each associated with different average values of reward and generosity throughout the experiment, as given in Table 2.

**Table 2.** Average generosity, reward, and point pool values for each of the four conditions.

Stimulus		Condition	Average generosity	Average reward	Average point pool
Human targets	Non-human targets				
Voice 1	Slot machine 1	High reward, low generosity	20%	20	100
Voice 2	Slot machine 2	Low reward, high generosity	40%	10	25
Voice 3	Slot machine 3	High reward, high generosity	40%	20	50
Voice 4	Slot machine 4	Low reward, low generosity	20%	10	50

Stimuli were rotated around these four conditions across participants.

Values for a target on a given trial were generated using the average reward/generosity value for that target’s condition (shown in Table 2) plus Gaussian noise (with *SD* = 10 for reward values and *SD* = 7.5 for generosity values). Specifically, normal distributions centred on these average values were created for each target condition, and trial by trial values generated by randomly sampling from these distributions. This random sampling came with the additional constraints that reward value be at least 2 points and generosity value be at least 1%. This was to ensure that the targets were never presented with reward values of 1 or 0 points, or generosity values of 0%; such values would have lacked meaning and been unhelpful for participants’ learning. The point pool for the chosen target on a given trial was then calculated by dividing the rounded reward value by the generosity value for that trial.

**Figure 1.**
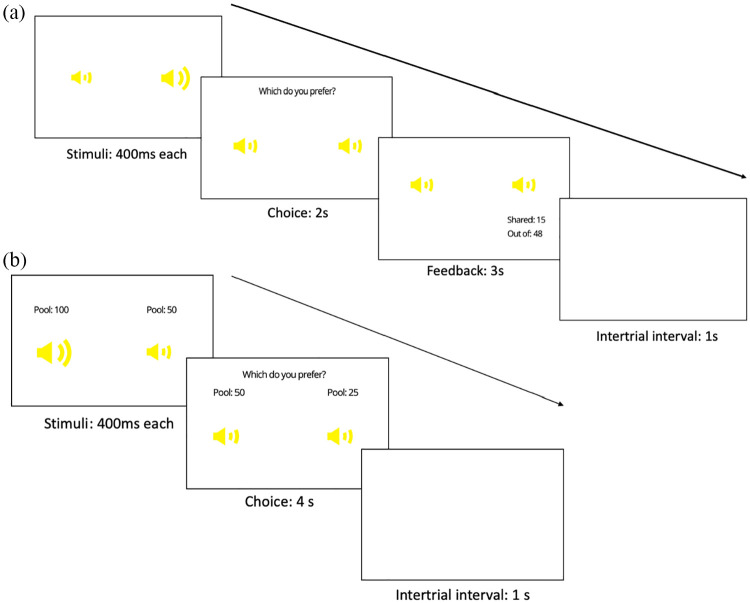
Trial procedure for (a) the training phase and (b) test phase. Yellow speakers would pulsate in turn (with order counterbalanced) to indicate location of each auditory target (left or right).

Participants first completed a training phase consisting of 72 trials broken up into 3 blocks. The structure of a training phase trial is outlined in Figure 1a. On each trial, a pair of auditory stimuli was presented, representing two of the targets. The tagged position of each auditory target (left or right) was indicated by the simultaneous pulsation of one of the speaker icon stimuli on screen (see section “Stimuli” for more details). The participant had to use a keyboard press to choose which target to play with on that trial (the left or right target), within a 2,000 ms time limit. A response was only possible after both stimuli had been played. After making their choice, feedback was given as to (1) the number of points that target chose to share with the participant (labelled as “Shared” for the human group and “Payout” for the slot machine group) and (2) the point pool available to the chosen target (labelled as “Out of” for both groups). These could be used to infer both the reward value (magnitude of points shared) and the generosity value (proportion of point pool shared). This feedback was presented for 3,000 ms. The number of points accrued so far by the participant was presented on screen at the end of each block. Both the tagged position of each target on the screen (left vs right) and the order of presentation of the targets (first vs second) were counterbalanced across trials. Furthermore, the trial-by-trial presentation of the stimuli and their associated reward/point pool values was randomised for each participant.

Following this training phase, participants completed a further 120 trials (divided into 5 blocks) in which they continued to make choices between pairs of targets. In this test phase however, participants were given information on the point pool available to each target before they made their choice, and received no feedback on the number of points shared after each trial, or the total points won so far at the end of each block (the total number of points won was given at the end of this phase). Participants had 4,000 ms to make their response. The trial structure for the test phase is outlined in Figure 1b. Point pools for each pair of targets in this phase were determined by first assigning a randomly generated integer between 10 and 100 to the first target. To generate the point pool for the second target, this amount was multiplied by one of seven ratios designed to be symmetrical around 1 (0.33, 0.67, 0.9, 1, 1.11, 1.5, and 3). Each pair was presented twice at every ratio, except for the 1:1 ratio at which they were presented 8 times. This was to allow for testing of fine-grained knowledge about the generosity values of targets.

Finally, participants completed a preference ratings task, in which they were asked to rate their liking of each of the four targets they had encountered in the previous tasks. Preference ratings were measured using a 7-point scale (1 = *not at all*, 7 = *very much*).

#### Data exclusion

In a first wave of data exclusion, whole datasets were excluded from participants who failed to achieve at least 75% accuracy on attention checks that were built into the above tasks. These consisted of infrequent (1 per block) and randomly occurring vigilance trials that required participants to make a specific keyboard press in response to an instruction presented onscreen, for example, “Press the M key.” Participants were also excluded who failed to follow the task instructions and took long breaks (more than 2 min) in between the training and test phases. Participants excluded according to these criteria were replaced, as outlined previously (see *Participants*). In a second wave of data exclusion, performance on individual trials was considered. Trials on which no response was made were excluded from data analyses. Participants who failed to respond on more than 20% of trials were excluded. Our pre-registered methods further stated that we would exclude trials on which reaction times were less than 200 ms, following the methods of Hackel et al. (2015); however, on examination of the data it was found that this resulted in exclusion of a large number of trials in most participants, suggesting that this exclusion rule was inappropriate. This is likely because our design differed from that of Hackel et al. (2015) in that a response was only possible after both stimuli had been presented. Instead, to ensure participants displaying extreme reaction times were removed, median test phase reaction times were calculated for each participant and those with median values more than 3 *SD*s below/above the group average were excluded. Based on this criterion, no participants from the current experiment were excluded, leaving a final sample size of 96 participants. The range of median reaction times in this sample was from 101.52 to 936.37 ms (*M* = 410.25 ms).

#### Hypotheses and statistical analyses

All data analyses were carried out using the statistical software R (R Core Team, 2019), except for the repeated-measures analysis of variance (ANOVA) carried out with the preference ratings data, which were conducted using SPSS. Analysis scripts and the data files for reproduction of these analyses can be found on the OSF page for this project (https://osf.io/yx3jt/). The hypotheses and statistical tests that were pre-registered for analysis of these data were chosen so as to replicate those reported in Hackel et al. (2015). The data were analysed to test four key hypotheses:

*Hypothesis 1.* Participants will be more likely to choose targets in the test phase that have been previously associated with both higher reward and higher generosity values in the training phase.

To test this hypothesis, we ran a multi-level logistic modelling analysis on the test phase data to predict the probability of choosing the left vocal target as a function of the difference in values (left minus right) for (1) point pool, (2) prior generosity value and (3) prior reward value. Prior generosity and reward values were calculated by taking the average reward/generosity associated with that target in the training phase. The left minus right differences for these three variables were then *z*-scored within-subjects so as to be on a similar scale. In addition to these predictors, we also added a fixed effect of target type (dummy coded as 1 for human and −1 for non-human) and a random effect of participant in the model. We predicted that the difference in generosity values and reward values would both be significant predictors of choice behaviour (probability of choosing the left vocal target). These analyses were carried out using the *lme4* package in R (Bates et al., 2015).

*Hypothesis 2.* Participants will show greater sensitivity to generosity value than reward value in such decisions.

To test this hypothesis, we contrasted the beta coefficients from the above multi-level modelling analyses for the reward and generosity difference value predictors using a *z*-test, with the prediction that the coefficient for generosity would be significantly greater than that for reward. This was done using the *esticon* function from the *doBy* package in R (Højsgaard & Halekoh, 2019).

*Hypothesis 3.* The effect of generosity will be even stronger for human targets than for slot machine targets.

To test this hypothesis, likelihood ratio tests were used to compare a model in which target type and generosity had additive effects with a model in which these two predictors showed an interaction. We predicted that the model with the interaction would provide a significantly better fit to the data.

*Hypothesis 4. This pattern of choice-making behaviour will show generalisation to subsequent ratings of preference for the same targets, such that*:
*(a) Participants will show higher ratings for human/slot targets previously associated with high generosity and high reward.*
*(b) The effect of generosity on these ratings will be greater than that of reward value for human targets only. Conversely for slot machines, the effect of reward value on these ratings will be greater than that of generosity.*

To test the first part of this hypothesis, we entered preference ratings into a 2 × 2 × 2 mixed-model ANOVA (generosity × reward × target type). First, we predicted a significant main effect of both generosity and reward. Furthermore, we predicted a reward by target type interaction, whereby reward value would have a stronger effect on ratings for the slot targets compared with the human targets, and a generosity by target type interaction, whereby generosity value would have a stronger effect on ratings for the human targets compared with the slot targets (demonstrated in simple effects analyses).

To investigate directly whether ratings for the human targets showed greater sensitivity to generosity or reward value, we calculated separate indices of reward sensitivity and generosity sensitivity as follows:

Reward sensitivity = average ratings for high reward targets—average ratings for low reward targets (collapsing across generosity value)

Generosity sensitivity = average ratings for high generosity targets—average ratings for low generosity targets (collapsing across reward value)

These sensitivity indices quantify for each individual the extent to which their ratings were driven primarily by the reward values of targets (without regard to generosity values), versus the generosity values of targets (without regard to reward values). These values were then compared for the human and slot machine groups separately by means of one-way repeated measures ANOVA. This was with the prediction that generosity sensitivity would be significantly greater than reward sensitivity for the human targets, but that the reverse would be true for the slot machine targets.

### Results

*Hypothesis 1*. Participants will be more likely to choose targets in the test phase that have been previously associated with both higher reward and higher generosity values in the training phase.

**Figure 2.**
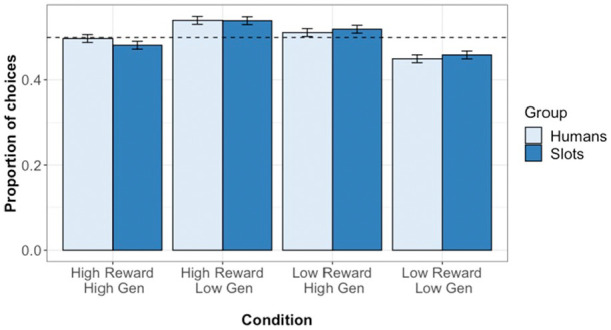
Test phase choice responses in Experiment 1. Plot shows the proportion of choices for which participants selected each condition. Error bars show standard error, while dashed line indicates chance (0.5).

The proportion of choices for which participants selected each condition is given in Figure 2. Multi-level logistic modelling analysis on test phase choice responses found a significant effect of pool difference (β= 0.616, *z* = 22.69, *p* < .001, *OR* = 1.852, CI = [1.757, 1.955]), and of prior reward difference (β = 0.042, *z* = 2.17, *p* = .03, *OR* = 1.043, CI = [1.004, 1.083]), but no significant effects of generosity (β = 0.016, *z* = 0.828, *p* = .224, *OR* = 1.016, CI = [0.978, 1.055]) or target type (β = 0.010, *z* = 0.486, *p* = .627, *OR* = 1.010, CI = [0.970, 1.052]).

*Hypothesis 2.* Participants will show greater sensitivity to generosity value than reward value in such decisions.

A linear contrast of the beta coefficients for the reward and generosity difference value predictors in the above multi-level modelling analysis found no significant difference between these, *t*(1) = 0.899, *p* = .343. Thus, the effect of generosity on test phase choices was not significantly greater than the effect of reward.

*Hypothesis 3.* The effect of generosity will be even stronger for human targets than for slot machine targets.

A likelihood ratio test comparing a model in which target type and generosity had additive effects with a model in which they showed an interaction did not find that the interactive model provided a significantly better fit to the data, χ^2^(1, 7) *=* 1.24, *p* = .266. Thus, the effect of generosity on test phase choices was not stronger in the human group than in the slot machine group.

*Hypothesis 4*. This pattern of choice-making behaviour will show generalisation to subsequent ratings of preference for the same targets, such that:
*(a) Participants will show higher ratings for human/slot targets previously associated with high generosity and high reward.*
*(b) The effect of generosity on these ratings will be greater than that of reward value for human targets only. Conversely for slot machines, the effect of reward value on these ratings will be greater than that of generosity.*

**Figure 3.**
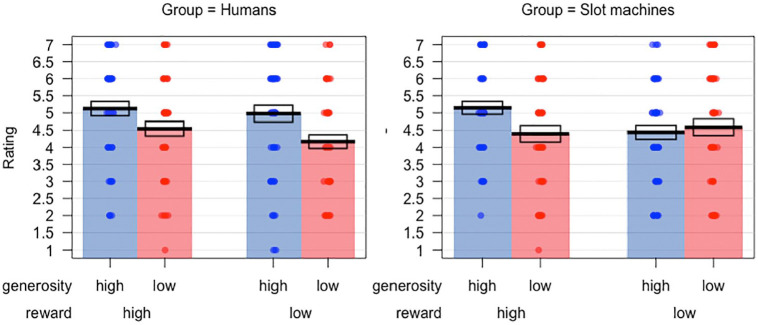
Preference ratings in Experiment 1 for the human and slot machine groups. Bars indicate means, while the boxes show the standard error of the mean.

Preference ratings in the two groups are plotted in Figure 3. A 2 × 2 × 2 mixed-model ANOVA on this data found a significant main effect of generosity, *F*(1, 94) = 9.509, *p* = .003, $\eta_{p}^{2}=.092$ , $\eta_{G}^{2}=.027$ = .027, but no main effect of reward or of group, and no significant interactions. One-way ANOVAs to compare reward and generosity sensitivity in the two groups found no significant differences for either the human targets, *F*(1, 47) = 1.5, *p* = .227, or for the slot machine targets, *F*(1, 47) = .016, *p* = .898.

### Interim discussion: Experiment 1

Overall, the results of this experiment present a mixed picture of whether reinforcement learning of rewards and traits can be demonstrated with auditory stimuli. Analysis of test phase data indicated that participants’ choices were significantly affected by the prior reward values of targets, suggesting successful learning of reward in the training phase. To our knowledge, this represents the first demonstration of successful reinforcement learning of rewards with auditory stimuli in a social learning paradigm. Reward value did not, however, appear to affect post-task ratings of liking for targets. Conversely, prior generosity of targets did not affect test phase choice behaviour, but did significantly affect post-task ratings. The expected interactions with target type were also not found; in particular, there was no evidence that generosity had a significantly greater effect on learning in the human group versus the slot machine group. This pattern of results thus fails to fully replicate findings reported by studies using visual stimuli in these paradigms, in which both reward and generosity effects were found for both test-phase choices and preference ratings (Familiar & Thompson-Schill, 2018; Hackel et al., 2015, 2020).

Previous work with visual stimuli has reported an effect of the “socialness” of the framing context on the relative weighting of reward and generosity in reinforcement learning (Hackel et al., 2015, 2020). In contrast, the current study did not find the expected interactions between generosity and target type, in which we had predicted a greater effect of generosity on learning in the human versus the non-human group. It is worth noting, however, that there were some suggestions of differences in responses to targets across groups in the preference ratings. From Figure 3, it can be seen that both groups show a clear effect of generosity when reward is high. However, when reward is low, the human group appears to rate high generosity targets more favourably than low generosity targets; this is not the case in the slot machine group, where high and low generosity targets are rated similarly when they yield low rewards. This trend, however, did not reach statistical significance. Therefore, in the current sample, the framing of the context as social or non-social did not have significant effects on learning.

In contrast to previous work with visual stimuli, we did not find any evidence of learning of traits in the test phase in the two groups. That is, the generosity behaviour of the targets in the training phase did not affect participants’ propensity to choose to play with them in the test phase. This questions whether reinforcement learning of traits can be demonstrated with auditory stimuli. Furthermore, learning about rewards did not transfer to target preference ratings, potentially suggesting that learning may be weak or non-transferable. There are multiple possible reasons why we failed to replicate the full pattern of learning of traits and rewards in this version of the reinforcement learning task. Broadly, these can be divided into (1) factors associated with adjustments made to the general design of the task and (2) factors associated with the auditory stimuli specifically. The first of these will be addressed in Experiment 2; the second will be discussed in the section “General Discussion.”

First, it is important to consider whether there are more general features of the current task design that may have caused this absence of effects. The key difference between the current paradigm and that used by Hackel et al. is the method of stimulus presentation on each trial. In their version of the task with visual stimuli, it was possible to (1) present both targets in a pair simultaneously with each other and (2) present feedback simultaneously with the selected target. Simultaneous presentation of the targets themselves allowed for minimal delay between the presentation and selection of a chosen target and the presentation of feedback. Furthermore, receiving the text feedback with the selected target still visible may have strengthened the formation of an association between the visual target and the feedback, facilitating better learning. Conversely, the major adjustment that was necessary for adapting the paradigm for use with auditory stimuli in the current study was to change the style of target presentation to sequential; to be heard clearly, each auditory target had to be presented on its own, and was not presented again at the time of feedback. This could have resulted in a greater delay between perception of the chosen stimulus and the feedback (particularly if the first stimulus was chosen).

Temporal contiguity is a key guiding principle in associative learning, both Pavlovian and instrumental (Allan & Church, 2002; Allan et al., 2003; Pavlov, 1927). In terms of conditioning procedures, our current design follows more closely a trace conditioning procedure (where there is a delay between the offset of the conditioned and the onset of the unconditioned stimulus); conversely, the design used by Hackel et al. (2015) with visual stimuli was more similar to a delay conditioning design (where the conditioned and unconditioned stimulus overlap in time and terminate together). In Pavlovian conditioning, research with non-human animals has reported that more trials are needed for the acquisition of associations through trace conditioning than delay conditioning (Beylin et al., 2001). Similarly, delaying feedback has been shown to impair learning during instrumental conditioning in non-human animals (Dickinson et al., 1992), and during perceptual classification tasks in humans (Maddox et al., 2003; Maddox & Ing, 2005).

Interestingly, there is evidence that such delays in feedback induce a shift in the underlying learning mechanism employed. Foerde and Shohamy (2011) presented patient and functional magnetic resonance imaging (fMRI) evidence that feedback delays induce a shift from striatal-based reinforcement learning towards episodic-based learning in the hippocampal system. In an associative learning task, Parkinson’s disease patients with damage to the striatum were found to demonstrate impaired learning with immediate feedback but intact learning with delayed feedback. Furthermore, healthy controls demonstrated increased activity in the ventral striatum when feedback was immediate, but increased activity in the hippocampus when feedback was delayed. Consistent with this, control participants’ episodic memory for feedback events was improved in the delayed feedback over the immediate feedback condition.

Overall, these previous findings suggest that delaying feedback may result in a shift in the balance of contributions of different underlying neural systems, which can cause a disruption or change in the nature of learning. It is therefore possible that the profile of learning effects observed in the current experiment was due to the task design necessarily introducing a greater delay between the presentation of the target stimuli and the feedback. This account fits particularly well with the finding of intact generosity effects in the preference ratings task; if learning is biased towards a hippocampal-based episodic learning mechanism, intact learning may be expressed through explicit ratings of target preference more reliant on recollection of target behaviour. This view fits less well, however, with the pattern of learning seen for reward, where effects were only seen for test phase choices.

To investigate whether the current failure to replicate the full pattern of learning effects with auditory stimuli could be explained by the use of sequential stimulus presentation, we ran a second experiment in which learning with sequential presentation of visual targets was examined. If such a set-up with visual stimuli showed a similar pattern of results, this would suggest that the pattern of learning seen with auditory stimuli was simply a result of the methodological design of the task. If, however, sequential presentation of visual targets yields a pattern of learning of reward and generosity that replicates previous work (e.g., Hackel et al., 2015), this would suggest that the results reported in Experiment 1 were specifically due to the use of auditory stimuli to represent the target identities.

## Experiment 2: effect of sequential presentation on trait learning with visual stimuli

### Method

#### Participants

A total of 109 participants were recruited for this experiment through the online recruitment platform Prolific. Data from 13 participants were excluded based on performance on attention checks and adherence to task instructions (see section “Data exclusion” from Experiment 1). After these exclusions, replacement participants were recruited to reach the target sample size of 96 participants (38 female, 57 male, 1 non-disclosed, mean age = 26.49, *SD* = 6.24). An equal number of participants took part in the two main conditions (48 in the human group, 48 in the non-human group).

#### Stimuli

**Figure 4.**
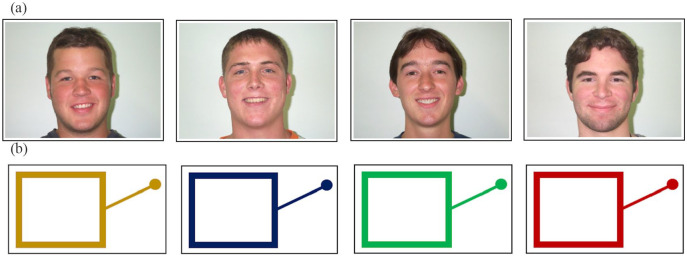
Visual stimuli for Experiment 2 used in the (a) human group and (b) slot machine group.

Visual stimuli consisted of four pictures of human faces (representing four human identities) and four line drawings of slot machines (representing four slot machine “identities”). The face stimuli consisted of pictures of four adult White male faces (see Figure 4a). These were identical to those used by Hackel et al. (2015) and were taken from the Park Aging Mind Face Database (Minear & Park, 2004). The slot machine stimuli consisted of schematic line drawings of slot machines in four different colours (see Figure 4b), based on stimuli used by Hackel et al. (2015). As before, participants only ever encountered the face stimuli (human group) or the slot machine stimuli (slot machine group). The same four pictures (one for each face identity/slot machine) were used throughout the whole experiment.

#### Procedure

The procedure and design were identical to those described in Experiment 1, but with the different target identities represented by the above described visual stimuli rather than the auditory stimuli. On each trial, the pictures were presented sequentially for 400 ms (to match the duration of the auditory stimuli in Experiment 1). All other timings were kept identical to Experiment 1.

#### Data exclusion

The same exclusion criteria from Experiment 1 were applied to data from this experiment. After replacement of participants who failed attention checks and did not adhere to task instructions (see section “Participants”), a further two participants were excluded due to extreme reaction times in the test phase (median reaction times more than 3 *SD*s below/above the group average). This left a total sample size of 94 participants whose data were used in analyses (48 in the human group, 46 in the slot machine group). The range of median reaction times in this sample was from 99.02 ms to 1,022.33 ms (*M* = 415.11). One participant in the human group failed to complete the preference ratings task, and so their data were included for the test phase only.

#### Hypotheses and statistical analyses

The same statistical analyses described for Experiment 1 were conducted with the data from Experiment 2, to investigate whether the predicted pattern of findings would be demonstrated with sequential presentation of visual stimuli. If the absence of certain expected significant effects of reward and trait learning with the auditory stimuli was due to the use of sequential presentation introducing a delay between stimulus presentation and feedback, we would expect sequential presentation with visual stimuli to produce similar results to those reported in Experiment 1. If, however, the absence of expected effects in Experiment 1 was specifically related to the use of auditory stimuli to represent target identities, we would expect to see robust effects with these visual stimuli that replicate previous findings with simultaneous presentation of visual stimuli.

### Results

*Hypothesis 1.* Participants will be more likely to choose targets in the test phase that have been previously associated with both higher reward and higher generosity values in the training phase.

**Figure 5.**
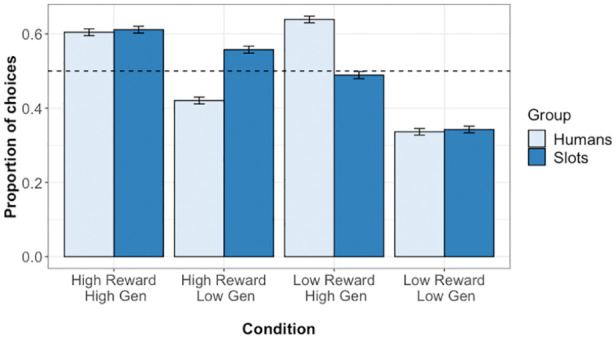
Test phase choice responses in Experiment 2. Plot shows the proportion of choices for which participants selected each condition. Error bars show standard error, while the dashed line indicates chance (0.5).

The proportion of choices for which participants selected each condition is given in Figure 5. Multi-level logistic modelling analysis on test phase choice responses found significant effects of pool difference (β = 0.711, *z* = 23.96, *p* < .001, *OR* = 2.036, CI = [1.922, 2.160]), of prior reward difference (β = 0.222, *z* = 10.99, *p* < .001, *OR* = 1.248, CI = [1.200, 1.299]), and of prior generosity difference (β = 0.479, *z* = 23.23, *p* < .001, *OR* = 1.616, CI = [1.552, 1.683]). There was, however, no significant effect of target type (β = −0.035, *z* = −1.31, *p* = .189, *OR* = 0.965, CI = [0.915, 1.018]).

*Hypothesis 2.* Participants will show greater sensitivity to generosity value than reward value in such decisions.

A linear contrast of the beta coefficients for the reward and generosity difference value predictors in the above multi-level modelling analysis found a significant difference, *t*(1) = 83.09, *p* < .001. Thus, the effect of generosity on test phase choices was significantly greater than that of reward.

*Hypothesis 3.* The effect of generosity will be even stronger for human targets than for slot machine targets.

A likelihood ratio test comparing a model in which target type and generosity had additive effects with a model in which they showed an interaction found that the interaction model provided a significantly better fit to the data, χ^2^(1,7) *=* 110.57, *p* < .001. As can be seen in Figure 5, this reflects the fact that generosity had a greater effect on choice responses in the human group than in the slot machine group.

*Hypothesis 4.* This pattern of choice-making behaviour will show generalisation to subsequent ratings of preference for the same targets, such that:
*(a) Participants will show higher ratings for human/slot targets previously associated with high generosity and high reward.*
*(b) The effect of generosity on these ratings will be greater than that of reward value for human targets only. Conversely for slot machines, the effect of reward value on these ratings will be greater than that of generosity.*

**Figure 6.**
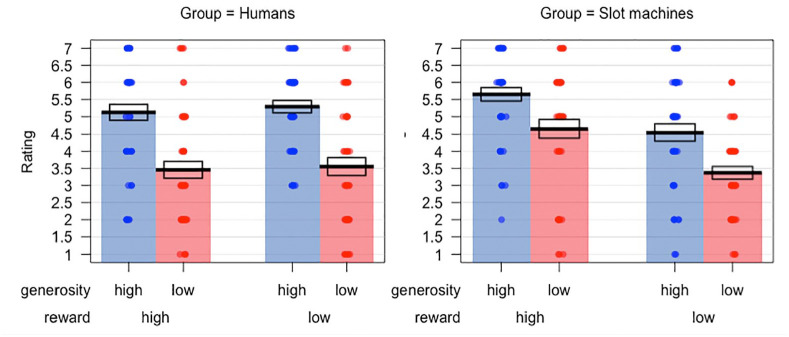
Preference ratings in Experiment 2 for the human and slot machine groups. Bars indicate means, while the boxes show the standard error of the mean.

Preference ratings in the two groups are plotted in Figure 6. A 2 × 2 × 2 mixed model ANOVA on these data found a significant main effect of generosity, *F*(1, 91) = 51.03, *p* < .001, $\eta_{p}^{2}=.359$ , $\eta_{G}^{2}=.166$ , and a significant main effect of reward, *F*(1, 91) = 12.12, *p* = .001, $\eta_{p}^{2}=.118$ , $\eta_{G}^{2}=.027$ , but no main effect of target type, *F*(1, 91) = 2.13, *p* = .148. There was, however, a significant interaction between target type and reward, *F*(1, 91) = 20.00, *p* < .001, $\eta_{p}^{2}=.180$ , $\eta_{G}^{2}=.044$ . This reflects a greater effect of prior reward on preferences in the slot machine condition than in the human condition. As can be seen in Figure 6, participants in the human condition show minimal discrimination between high and low reward targets in their ratings.

A one-way ANOVA comparing generosity sensitivity and reward sensitivity in the human group found a significant difference, in which ratings were more sensitive to generosity than to reward, *F*(1, 46) = 39.84, *p* < .001, $\eta_{p}^{2}=.464$ , $\eta_{G}^{2}=.224$ . The same analysis in the slot machine group, however, found no significant difference between reward and generosity sensitivity.

### Interim discussion: Experiment 2

Overall, the pattern of results found in Experiment 2 using sequential presentation of visual stimuli was very similar to that previously reported in reinforcement learning paradigms using simultaneous presentation (Hackel et al., 2015, 2020). Participants demonstrated significant learning about reward and trait outcomes, and this learning was biased by the social framing of the context; specifically, generosity information was prioritised for learning with human targets but not with non-human targets. Therefore, the use of sequential presentation with visual stimuli did not appear to drastically alter the pattern of reinforcement learning of traits and rewards from what has been previously reported in the literature.

This successful replication of learning effects in a sequential presentation version of the task is not incompatible with the suggestion that a change in the underlying neural mechanism supporting learning could have occurred. As discussed previously, research on the effects of delays on reinforcement learning would predict that the sequential presentation would induce a shift from reliance on striatal-based learning to reliance on the hippocampal system. However, such a shift in learning mechanism need not always result in a detriment in performance; in the study by Foerde and Shohamy (2011) control participants showed no difference in performance for learning cue-outcome associations with immediate versus delayed feedback, despite the changes in underlying neural activity. Thus, for learning with sequential presentation of visual targets, these complementary neural learning mechanisms may have been able to sustain equivalent levels of performance in the task.

One potential area of difference in learning patterns observed with the current sequential presentation paradigm is that learning with human targets appeared to be particularly strongly biased towards generosity information. Specifically, there was more limited learning of reward outcomes in the human condition than previously observed, in both test phase choices and preference ratings (see Figures 5 and 6). Such biased learning has been reported in previous versions of the task (Familiar & Thompson-Schill, 2018; Hackel et al., 2015, 2020), however, it is interesting that the current paradigm appeared to yield an exaggerated generosity bias with human targets. Why the use of sequential presentation of stimuli would have increased the weighting of learning towards generosity for human targets is, however, unclear. One possibility is that sequential presentation in the test phase allowed more time for this enhanced knowledge about the prior generosity of targets to be expressed.

Overall, the results from Experiment 2 largely replicate previous findings from studies using visual stimuli in social reinforcement learning paradigms (Hackel et al., 2015, 2020). Specifically, the results demonstrate that significant reinforcement learning of trait and reward information can occur with sequential presentation of visual stimuli, and that learning appears biased by the social framing of the context. Thus, the introduction of a small delay between stimulus presentation and feedback is not sufficient by itself to disrupt reinforcement learning with visual stimuli.

## General discussion

The aim of the current study was to investigate whether reinforcement learning of rewards and traits could be demonstrated with auditory stimuli. Using a social learning task with auditory target identities, we failed to replicate patterns of learning of reward and trait outcomes previously reported with visual targets (Experiment 1). When replicating this task design with visual stimuli (Experiment 2) we were able to replicate previously reported patterns of learning, including interactions with the animacy of the targets. This suggests that the failure to demonstrate the full expected pattern of learning with auditory stimuli in Experiment 1 cannot be completely explained by the sequential presentation of targets. Thus, although we did find some evidence of successful reinforcement learning of rewards with auditory stimuli, the general pattern suggests that the use of auditory stimuli may have affected learning of rewards and traits in this paradigm.

### Discriminability of the auditory stimuli

One interpretation of the failed replication of learning patterns in Experiment 1 is that this reflects something about the specific characteristics of the particular auditory stimuli that were used. For example, the different auditory identities may not have been sufficiently discriminable to allow targets to be robustly mapped onto representations of rewards and traits. All voices were matched on sex and accent, which may have made it difficult for participants to reliably tell the different vocal identities apart. It is possible that the use of voices that were more distinctive from one another would have facilitated learning of the different identities, and thus learning of their different reward and generosity values.

However, increasing the distinctiveness of the voice stimuli could itself interfere with learning from feedback. People readily form personality impressions from mere exposure to voices alone (McAleer et al., 2014) and there is evidence that these first impressions can interact with learning about the behaviour of those agents (Torre et al., 2018, 2020). Changing the sex, accent, or even just the pitch of the different vocal identities would likely have resulted in differences in their initial perceived attractiveness or trustworthiness, which could have biased learning about their behaviour. To avoid such issues, the voice stimuli in the current experiment were matched on ratings of trustworthiness, attractiveness, and likeability. Thus, if we aim to model learning from well-controlled yet naturalistic voice stimuli, it is practically not possible to simultaneously ensure that the identities they represent are maximally discriminable. It should be further noted that the chances of successful voice identity discrimination in the current study were increased by the use of single tokens for each identity; when learning voice identities in a single speaking style (e.g., read speech) it has been shown that training with low variability stimulus sets is more beneficial than high variability stimulus sets (Lavan et al., 2019).

Finally, perhaps the strongest argument against this discriminability explanation is that it does not account well for the intact learning of reward values in the test phase. That is, the auditory stimuli must have been sufficiently discriminable to allow significant learning of reward values to guide test phase choices. Furthermore, the pattern of learning was the same for both voices and slot machine tone stimuli. As simple tone sequences, the slot machine sounds would have been more easily discriminable than the voices, and yet there was no evidence for greater reinforcement learning of traits in the non-human than the human condition.

### Intact learning of traits in auditory target preference ratings

A pertinent question in this discussion concerns why participants in Experiment 1 were able to demonstrate learning about the generosity of targets in their explicit ratings of preference, despite the absence of generosity effects on their test-phase choice responses. Conversely, expression of reward learning was limited to test phase choices, and did not filter through to participants’ explicit ratings of liking. This presents a picture in which patterns of learning about rewards and traits appear to have been differentially affected by the use of auditory stimuli.

It is of interest to consider by what mechanism participants in Experiment 1 were able to form explicit trait impressions. Previous findings reported by Hackel et al. (2015, 2020) were used to argue that attitude formation can occur via reinforcement learning. However, the current absence of generosity effects in the auditory test phase data casts serious doubt on whether any reinforcement learning of traits had occurred. This suggests that the preferences participants came away with must have been formed via some other mechanism. This fits with predictions from previous work on the effect of delay on instrumental learning; as previously discussed, this is proposed to induce a shift from reliance on striatal-based reinforcement learning to reliance on hippocampal-based episodic learning (Foerde & Shohamy, 2011). In Experiment 1, reliance on this latter learning mechanism may have thus resulted in the formation of explicit trait judgements (in episodic memory) that were not available to guide implicit choice responses in the test phase.

Conversely, the reverse pattern of learning for reward was found, in which participants chose to play with auditory targets previously associated with higher reward values, but did not rate these more highly when asked about their explicit preferences. This suggests that learning of reward values remained more implicit, and perhaps did not show the same shift to a reliance on more explicit hippocampal-based learning. It is difficult to know why this would be the case; however, it is worth considering that while reward values were directly perceptible to participants in feedback, generosity values had to be inferred from that feedback (i.e., through a mental calculation of reward value divided by point pool). This extra step may have encouraged more explicit processing of the generosity of targets, while immediately available reward values could be more easily incorporated into implicit associations via reinforcement learning. This remains speculative, but suggests that further work is needed to consider the mechanisms underlying learning of traits versus rewards in these paradigms.

These ideas could be tested by adjusting the feedback in the training phase to include explicit information about the generosity of targets. For example, in addition to telling participants the number of points shared by a target and their available point pool, one could also provide the corresponding percentage of points shared (i.e., a direct measure of target generosity). By removing the need for additional mental calculations in working memory, this may boost implicit trait learning with auditory targets to result in significant generosity effects for the test phase.

### Impact of working memory demands on learning

In Experiment 2, however, learning of both reward and generosity was seen with visual stimuli for both the test phase and preference ratings. This suggests that the potential difficulties associated with the mental calculation of generosity values—as discussed above—need not always be an impediment to learning. This may instead be further dependent on the type of target stimuli this information is to be associated with. Specifically, it may be particularly difficult to combine such mental calculations about generosity with dynamic auditory stimuli that unfold over time, due to working memory limitations. Furthermore, such mental calculations would be likely to activate the “inner voice” of the participant; this could then potentially interfere with the representation of the target voice being maintained in working memory.

Multiple studies have reported a more limited capacity to store auditory than visual stimuli in short term memory, which is further exacerbated by longer retention delays (Bigelow & Poremba, 2014; Cohen et al., 2009). For auditory stimuli, playing of distractor stimuli during the retention interval has also been shown to have a particularly disruptive effect on retention (Berman et al., 2009; Pechmann & Mohr, 1992). The sequential presentation of two auditory targets in the current task may thus have meant that a non-chosen auditory stimulus interfered with the memory of the chosen auditory stimulus, thus weakening its representation and the ability for it to become associated with the feedback. As noted above, for trait learning, the mental calculation of generosity values would have placed further demands on working memory, exacerbating this problem.

This difference in working memory demands between auditory and visual versions of the task is most apparent for stimuli in the slot machine condition. In Experiment 2, this condition involved slot machine icons that could be differentiated on the basis of colour, implicitly providing verbal labels for each of the targets (e.g., “Red,” “Green”). These may have been easier to encode and rehearse in working memory, enabling recruitment of the phonological loop (Baddeley, 2000; Baddeley & Hitch, 1974, 2019). Conversely, although the auditory slot machine stimuli were relatively simple and easily discriminable, it is likely that representing and maintaining such tone sequences would have been more difficult, unless perhaps the listener was musically trained (Cohen et al., 2011). These differences in working memory demands could thus underlie the differences in the apparent extent of learning between these auditory and visual conditions.

It is worth pointing out, however, that the “auditory” condition does in fact contain both visual and auditory stimuli; the voices and slot machine tone sequences are accompanied by pictures of speakers which pulsate to indicate the position (left versus right) of each target. These speakers are similar to the visual stimuli used for the slot machine condition in Experiment 2, in that they are simple coloured line drawings. It is therefore possible that these visual stimuli were incorporated into the associations formed with reward and generosity values; as these were identical across targets, this may have thus dampened discrimination between the different conditions. It would be challenging, however, to circumvent the need for these visual stimuli while keeping the design of the task similar to that of the visual condition.

One possible modification of the auditory version of the task in Experiment 1 could be to use voice stimuli that say different words; for example, rather than all voice tokens saying “Hello,” each voice could be assigned a different greeting such as “Hi,” “Hey,” “Hiya” and “Hello.” This could enable the use of well-matched stimuli (in terms of accent and other variables affecting rapid personality impressions) that would be highly discriminable, and crucially that could be assigned a verbal label to facilitate better encoding and rehearsal in working memory. We would predict that such adjustments would strengthen learning of reward and trait outcomes during reinforcement learning with auditory stimuli. However, it should be noted that such a modification would deviate from the question of whether participants can use reinforcement to learn to associate trait information with specific voices per se, rather than with the verbal content of those voices.

## Summary and conclusion

In sum, the current study provides an important demonstration of successful reinforcement learning of rewards with auditory stimuli in a social learning task; however, the pattern of learning did not fully replicate that previously reported in equivalent paradigms using visual stimuli. Conversely, the expected pattern of learning effects could be demonstrated when replicating the same paradigm with visual targets, suggesting that sequential presentation of stimuli need not necessarily interfere with learning of reward and trait outcomes. We suggest that the compounding effects of the (necessary) sequential presentation of targets and reduced working memory capacity for auditory stimuli may have placed severe limitations on the extent of implicit associative learning that could occur for trait information. These constraints may have had a less severe effect on reinforcement learning of reward values, as these were more immediately available from feedback. Conversely, some explicit learning of preferences based on trait inferences appears possible, which may be mediated through reliance on the hippocampal system for learning. Overall, more work on reinforcement learning with auditory stimuli is needed, to consider how the mechanisms underlying learning of reward versus trait information may differ.
